# The Efficacy and Toxicity of Paclitaxel Plus S-1 Compared With Paclitaxel Plus 5-Fu for Advanced Gastric Cancer: A PRISMA Systematic Review and Meta-analysis of Randomized Controlled Trials

**DOI:** 10.1097/MD.0000000000000164

**Published:** 2014-11-28

**Authors:** Huan Liu, Xiaowan Chen, Jingxu Sun, Peng Gao, Yongxi Song, Ning Zhang, Xiaoli Lu, Huimian Xu, Zhenning Wang

**Affiliations:** From the Department of Surgical Oncology and General Surgery, First Hospital of China Medical University (HL, XC, JS, PG, YS, XL, HX, ZW) and Department of Pathophysiology, School of Basic Medical of China Medical University (NZ), Shenyang, China.

## Abstract

The standard treatment for patients with advanced gastric cancer (AGC) is still a matter of debate. The chemotherapy regimen of paclitaxel (PTX) combined with S-1 has been used to treat AGC or metastatic gastric cancer.

We conducted a meta-analysis to compare oral S-1 and infusional 5-fluorouracil (5-FU) to determine which agent was more efficacious and less toxic in combination with PTX. A systematic review with a meta-analysis was performed. PubMed, EmBase, the Cochrane Central Register of Controlled Trials, and the China National Knowledge Infrastructure databases were searched to select randomized controlled trials (RCTs) comparing PTX plus S-1 and PTX plus 5-FU in patients with AGC.

Three RCTs were eligible and 352 patients were analyzed. PTX plus S-1 increased the disease control rate (risk ratio [RR] = 1.14, 95% confidence interval [CI] = 1.00–1.30, *P* = 0.04) and reduced the progressive disease rate (RR = 0.62, 95% CI] = 0.39–0.98, *P* = 0.04) compared with PTX plus 5-FU. There was a significant decrease in nausea (RR = 0.60, 95% CI = 0.43–0.82, *P* = 0.001) and vomiting (RR = 0.55, 95% CI = 0.33–0.91, *P* = 0.02) in patients treated with PTX plus S-1.

PTX plus S-1 was associated with almost equivalent safety and a lower progressive disease rate compared with PTX plus 5-FU. PTX plus S-1 is a good alternative strategy for patients who cannot tolerate a continuous intravenous infusion.

## INTRODUCTION

Gastric cancer (GC) is the second leading cause of cancer deaths and the fourth most common cancer worldwide^1,2^ with a particularly high incidence rate in Asia.^3^ GC is very difficult to cure because GC is often not detected until at an advanced stage. Even with the best supportive care, the median survival time for patients with nonresectable or metastatic GC is only 3.1 months.^4^ For advanced gastric cancer (AGC), combination chemotherapy has demonstrated a survival benefit compared with best supportive care.^4,5^ However, Standard treatment for AGC is still controversial. 5-Fluorouracil (5-FU)-based regimens have long been used in first-line treatment of AGC. The development of new chemotherapeutics is underway to improve the outcome of patients with AGC. Among some newly developed chemotherapeutic drugs, paclitaxel (PTX) and S-1 have received considerable attention from researchers. S-1 is a new oral fluorouracil drug consisting of tegafur and 2 modulating agents (gimeracil and potassium oxonate) at a molar ratio of 1:0.4:1.^6^ Phase II trials of S-1 therapy for AGC conducted in Japan have shown a high overall response rate of 44% to 54%.^7,8^ And it has been confirmed that fluoropyrimidine is synergistic with taxane.^9,10^ The response rate of PTX monotherapy for AGC is 20% to 25%, and is not affected by the degree of differentiation of adenocarcinoma.^11,12^ PTX has excellent pharmacokinetics and antitumor effects on the peritoneal dissemination of GC.^13^ Some studies have reported that PTX plus S-1 is a feasible and effective regimen for chemotherapy in patients with AGC.^14,15^ The traditional infusion of 5-FU plus PTX has also been studied^16,17^; however, whether or not S-1 is optimal in combination with PTX for AGC remains to be confirmed.

We conducted a meta-analysis to compare S-1 with infusional 5-FU to determine which agent is superior in combination with PTX with respect to efficacy and toxicity in patients with AGC.

## METHODS

### Literature Search Strategy

We searched PubMed, EmBase, the Cochrane Central Register of Controlled Trials, and the China National Knowledge Infrastructure Database up to November 31, 2013. The search terms included “paclitaxel,” “S-1,” “stomach neoplasms,” “fluorouracil,” “gastric cancer,” ‘carcinosis,” and “randomized trial” combined with AND/OR. The search also included all of the mesh terms. No search restrictions were imposed. The reference lists of all retrieved articles were reviewed for further identification of potentially relevant studies. Review articles were also obtained to identify other possible studies.

### Study Selection

Clinical trials that met the following criteria were included in the meta-analysis, as follows: patients diagnosed with AGC; trials comparing PTX plus S-1 and PTX plus 5-FU; and randomized controlled trial (RCT); mandatory reporting of survival outcomes, response rates, and toxicities. Excluded criteria: nonrandomized prospective and retrospective comparative trials; trails unable to get all of the data; and duplicate publications.

Furthermore abstracts of RCTs presented at national and international meetings were also excluded to prevent the duplication of data. Duplicate published trials with accumulating numbers of patients or increased lengths of follow-up were considered in the last or at least the more complete version.

Two independent reviewers (HL and XC) assessed each study for inclusion using a standardized form with eligibility criteria and cross-checked to reach consensus. Each study was fully examined to eliminate duplicates.

### Data Extraction and Quality Assessment

Two independent investigators (HL and XC) reviewed the publications, extracted the data, and reached a consensus on all items. The data collected for each study included the following: basic information from articles, such as year of publication, country, and names of authors; characteristics of patients, such as age and sex; information of study, such as sample size, study design, randomization scheme, inclusion criteria, and type of end point used; and information of treatment, such as treatment regimens, median overall survival (OS), progression-free survival (PFS), time-to-failure (TTF), time-to-progression (TTP), overall response rate (ORR), disease control rate (DCR), and toxicities (grade I–IV). ORR was defined as the percentage of patients with a complete or partial response. DCR was defined as the percentage of patients with a complete or partial response or stable disease. The response rate was based on the Response Evaluation Criteria in Solid Tumors (RECIST) criteria and toxicity was graded according to the National Cancer Institution Toxicity Common Criteria. The available information was extracted and recorded on a data collection form and entered into an electronic database. The quantitative 5-point Jadad scale was used to assess the quality of included trials based on the report of the methods and results of the studies.^18^

### Statistical Analysis

The Mantel–Haenszel (M-H) test was used for comparison of dichotomous data and the risk ratio (RR) or risk difference (RD) estimate. The 95% confidence interval (CI) of the RR or RD was also calculated. A statistical test with a *P* value <0.05 was considered significant. We assessed for heterogeneity in summary effects using the Cochrane Q and the I^2^ test (with 95% CIs). We considered a *P* value <0.05 and I^2^ ≥50% to indicate significant heterogeneity. If heterogeneity existed,^19^ data were analyzed using a random-effects model, and a subgroup analysis was used to explore sources of heterogeneity. In the absence of heterogeneity, a fixed-effects model was used. The statistical analysis was performed by Review Manager (RevMan) [Computer program]. Version 5.2. Copenhagen: The Nordic Cochrane Centre, The Cochrane Collaboration, 2013.

## RESULTS

### Literature Search and Selection

**FIGURE 1:**
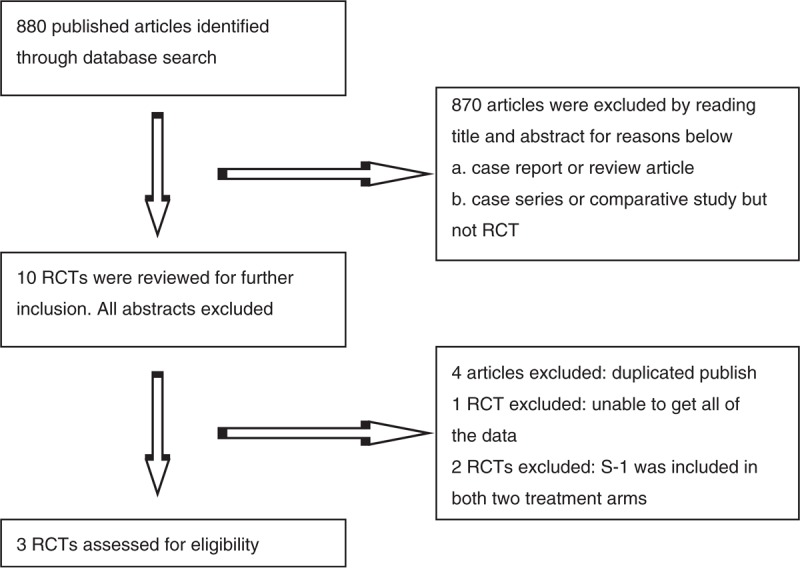
Flow chart of trial selection process. RCTrandomized controlled trial

The literature search and selection procedure are shown in Figure 1. Three RCTs^20–22^ were eligible for analysis and 352 patients with nonresectable, palliative-resected, recurrent, or metastatic GC were included: 182 patients in PTX plus S-1 group and 170 patients in PTX plus 5-FU group (Table 1). The sample size of individual RCTs ranged from 44 to 229. There were no significant differences in the baselines between the PTX plus S-1 and control groups in these studies, as reported.

**TABLE 1:**
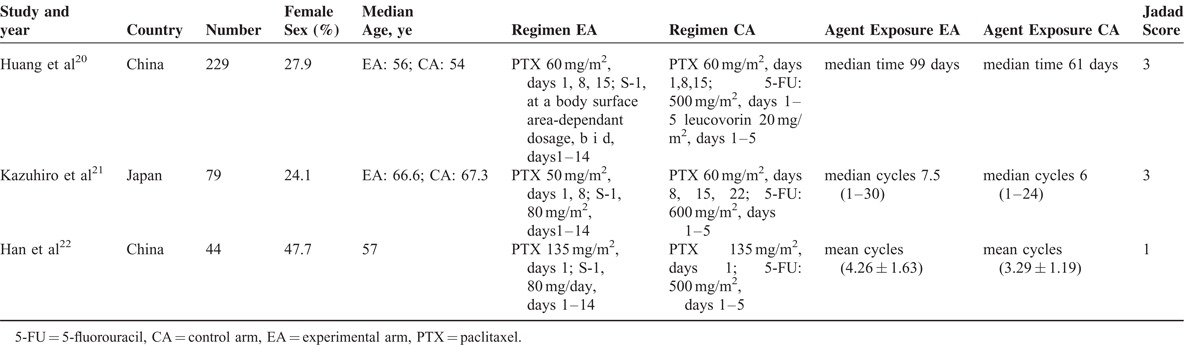
Overview of Studies in the Pooled Analysis (N = 352)

### Response

**FIGURE 2:**
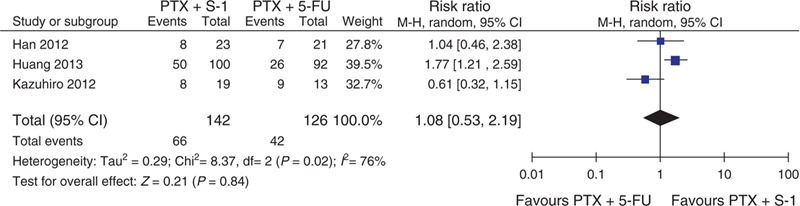
Forest plot of ORR. 5-FU5-fluorouracil, CI = confidence interval, ORR = overall response rate, PTX = paclitaxel.

**FIGURE 3:**
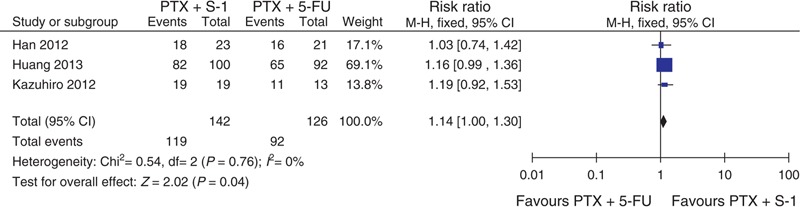
Forest plot of DCR. 5-FU = 5-fluorouracil, CI = confidence interval, DCR =  disease control rate, PTX = paclitaxel

All 3 studies assessing 268 participants who were randomized to receive PTX plus S-1 (n = 142) or PTX plus 5-FU (n = 126) provided the information on complete response (CR), partial response (PR), stable disease (SD), and progressive disease (PD). The ORR (combined CR and PR) was 46.5% (66/142) versus 33.3% (42/126) in the PTX plus S-1 and PTX plus 5-FU regimens, respectively. This meta-analysis showed that there was no significant difference between these 2 groups (RR = 1.08, 95% CI = 0.53–2.19, *P* = 0.84; Figure 2). The DCR (combined of CR, PR, and SD) was 83.8% (119/142) versus 73.0% (92/126) in the PTX plus S-1 and PTX plus 5-FU regimens, respectively. This meta-analysis (RR = 1.14, 95% CI = 1.00–1.30, *P* = 0.04) showed that there was a significantly better DCR with the PTX plus S-1 regimen (Figure 3). The meta-analysis results of CR, PR, SD, and PD were listed in Table 2. The PD rate was significantly different between the 2 groups.

**TABLE 2:**
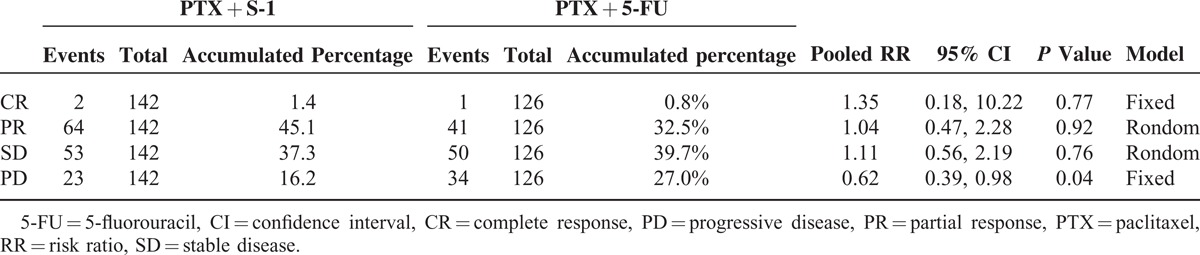
Response Comparison Between PTX + S-1 and PTX + 5-FU Chemotherapy

### Survival Outcomes

With respect to PFS and TTF, Huang et al^20^ showed that the median PFS of the experimental and control arms was 153 and 129 days, respectively (hazard ratio [HR] = 0.641, 95% CI = 0.473–0.868, *P* = 0.004). These results were significantly different, which indicated a favorable outcome in the PTX plus S-1 group for PFS. The median TTF of the 2 arms was not reported. The HR of TTF in the 2 arms was 1.449 (95% CI = 0.705–2.980, *P* = 0.229), which indicated there was no significant difference. The 6-month PFS rates in both the arms were similar (31.3% vs 31.8%, *P* = 0.94).

Only 1 RCT has reported a 10-month OS rate and median survival time (MST).^21^ The trial had 4 arms; only arms C (PTX plus 5-FU) and D (PTX plus S-1) were adopted. The 10-month OS rates were 61% and 73% in arms C and D, respectively. The MST values were 410 and 462 days in arms C and D, respectively. Kaplan–Meier survival curves did not show a significant difference between the 2 arms.

Han et al^22^ reported that the median TTPs were 6.5 and 5.5 months in the PTX plus S-1 and PTX plus 5-FU groups, respectively. There was no significant difference in TTP values in the 2 groups by log-rank test (*χ*^2^ = 0.13, *P* = 0.714).

### Toxicities

We compared grade I - IV and grade III - IV toxicities in both arms according to reported information. Most of the toxicities were hematologic and gastrointestinal in nature. Hematologic toxicities, including leucopenia, neutropenia, thrombocytopenia, and anemia, were not significantly different in the 2 groups. There was a significant increase in nausea (grade I - IV: RR = 0.60, 95% CI = 0.43–0.82, *P* = 0.001) and vomiting (grade I - IV: RR = 0.55, 95% CI = 0.33–0.91, *P* = 0.02) with the PTX plus 5-FU regimen. No significant differences were detected with respect to other toxicities. One trial reported treatment-related deaths; 1 patient in the experimental arm died of neutropenia and infection, whereas 1 patient in the control arm died of upper gastrointestinal haemorrhage.^20^ The meta-analysis results of these toxicities are listed in Table 3.

**TABLE 3:**
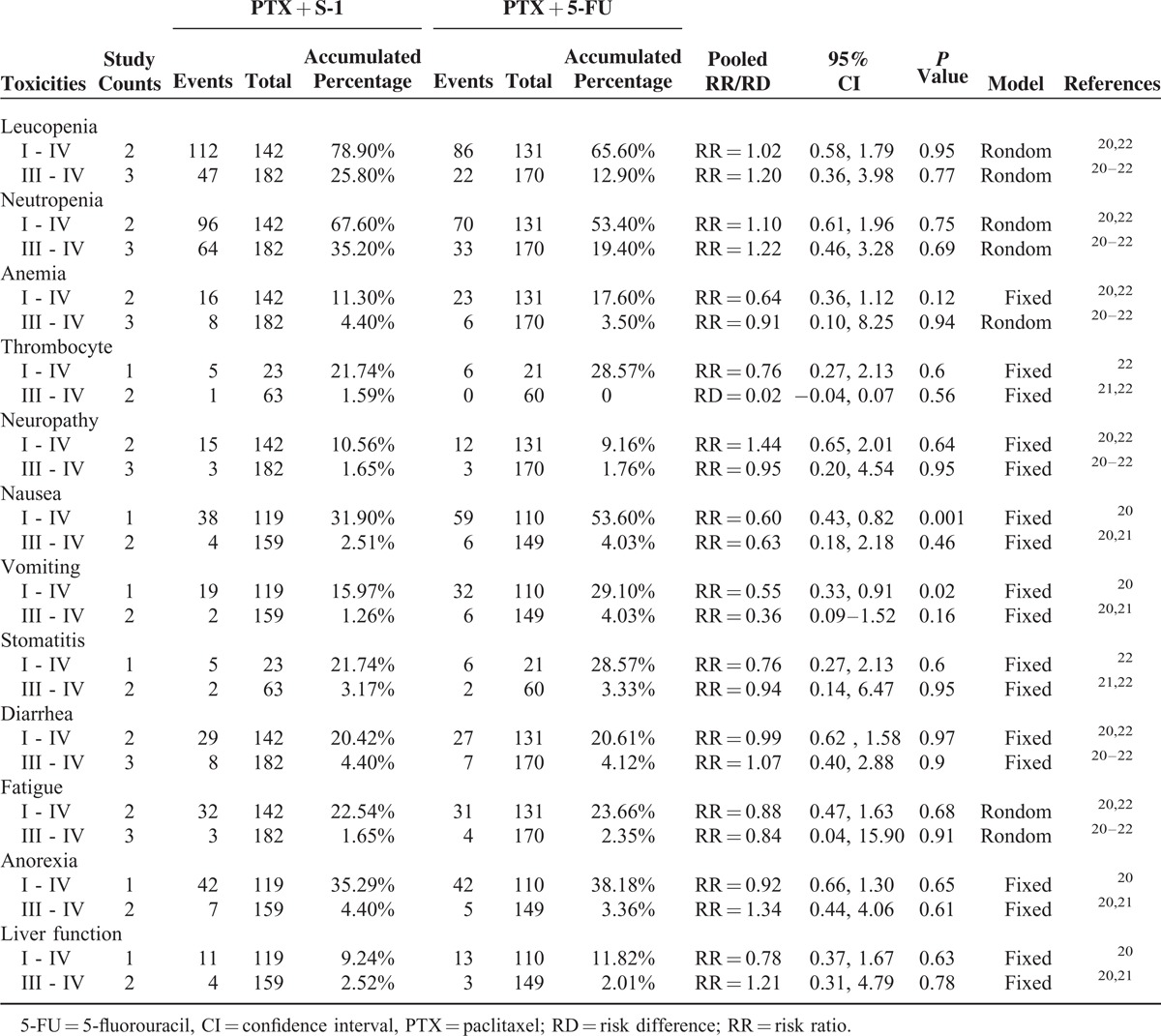
Toxicities Comparison Between PTX + S-1 and PTX + 5-FU chemotherapy

### Publication Bias

**FIGURE 4:**
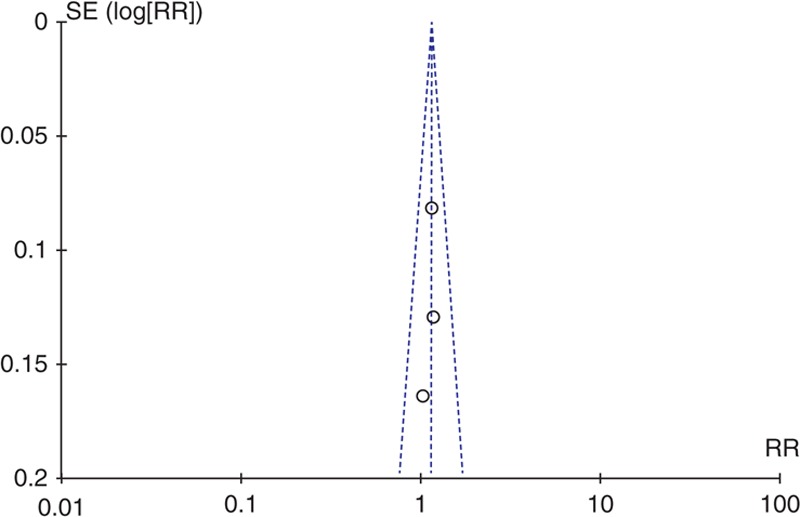
Funnel plot. RR = risk ratio.

A funnel plot was generated to assess the publication bias of the literature. The funnel plot showed that there was no apparent publication bias (Figure 4).

## DISCUSSION

Although GC is a relatively chemosensitive disease with response rates of 30% to 40%, chemotherapy in patients with AGC is limited by a low CR rate, response durability that is short-lived, and considerable toxicity.^23^ For many years, 5-FU-based regimens have long been used in first-line treatment of AGC. A highly effective and well-tolerated regimen is needed. Among the recently emerging new drugs and therapies, we focused on PTX combined with S-1. A phase II randomized trial reported by Mochiki et al showed that PTX combined with S-1 is a feasible and effective non-platinum-based regimen for chemotherapy in patients with AGC.^15^ Research on the efficacy of traditional regimens and infusion of 5-FU with PTX was ongoing simultaneously^16,17^ Hence, we undertook a meta-analysis of published data from RCTs to determine whether or not oral administration of S-1 is a good alternative agent for infusional 5-FU with PTX for AGC. A systematic review of the literature revealed 3 eligible RCTs with 352 patients, all of which had compared the efficacy of PTX plus S-1 with PTX plus 5-FU.

The meta-analysis showed a slightly better disease control rate in patients who received PTX plus S-1. Pooled analysis also showed that PTX plus S-1 therapy is superior to PTX plus 5-FU with respect to the ORR; however, the improvement was not significant. A higher progressive disease rate characterized the PTX plus 5-FU group. In recent years, there have been several studies involving PTX plus S-1 regimen in patients with AGC. Wang et al^24^ reported the ORR and median PFS of PTX plus S-1 therapy to be 46.3% and 6.0 months, respectively. Inada et al. reported the ORR and median PFS of PTX plus S-1 to be 55% and 4.7 months, respectively.^25^ The results of the two trials were comparable with the results reported herein.

We could not perform a pooled analysis on survival outcomes because the 3 trials evaluated prognosis with variant indicators. The majority of survival outcomes did not show a significant difference between the 2 groups in these 3 trials, except Huang's study on PFS,^20^ which indicated that patients who received PTX plus S-1 had a superior PFS. From the data reported by the included 3 studies, we considered that PTX plus S-1 regimen was not inferior to PTX plus 5-FU regimen with respect to survival outcomes at least.

The results of our study also showed that the toxicities between the 2 groups were almost equivalent; there was no significant difference between the 2 groups with respect to grade III - IV toxicities. However, with respect to grade I - IV toxicities, there was a significant decrease in nausea and vomiting in patients treated with PTX plus S-1. Other studies^15,24^ have suggested that PTX plus S-1 is well-tolerated, which is in agreement with our findings.

We noticed that patients in the PTX plus S-1 group had a longer exposure time or completed more chemotherapy cycles in all 3 trials (Table 1). This phenomenon reflected the superiority of PTX plus S-1 versus PTX plus 5-FU with respect to treatment compliance. The clinical outcomes of treatment are affected not only by how well patients take their medications, but also by how long patients take their medications.^26^ Considering the poor prognosis of AGC, application of an effective, well-tolerated, and more convenient regimen is particularly important. Generally speaking, treatment with an oral agent would be preferable for patients and medical staff than a treatment requiring a continuous intravenous infusion, which has risks of infection, thrombotic events, or other side effects.

**FIGURE 5:**
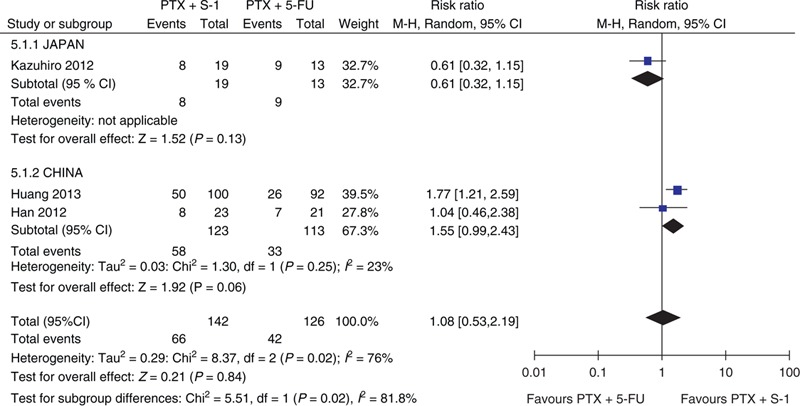
Forest plot of ORR Subgroup analyzes by area. 5-FU = 5-fluorouracil, CI = confidence interval, M-H = Mantel–Haenszel, ORR = overall response rate, PTX = paclitaxel

Heterogeneity existed in the incidence of the ORR to PTX plus S-1 and PTX plus 5-FU in the treatment of AGC. Analysis was performed under a randomized effects model. Subgroup stratification analysis showed that sources of heterogeneity were appraised by area (Figure 5). The results of subgroup stratification analysis indicated the heterogeneity associated with the differences between the two countries. Differences amongst these three trials, such as age, gender, doses, and regimen of therapy were also considered; however, due to the limited data, we did not conduct subgroup stratification.

This is the first pooled analysis of PTX plus S-1 compared with PTX plus 5-FU for AGC to date. Huang et al^27^ conducted a similar meta-analysis of S-1-based therapy versus 5-FU-based therapy for patients with AGC, but they did not assess PTX plus S-1 and PTX plus 5-FU. The therapies included in their study included S-1 monotherapy, 5-FU monotherapy, S-1 plus cisplatin therapy, and 5-FU plus cisplatin therapy, but did not include PTX plus S-1 therapy and PTX plus 5-FU therapy. The Huang et al study showed that S-1-based therapy is associated with a better OS and a near-equivalent ORR and safety profile compared with 5-FU-based therapy. The results were similar to the results reported herein.

There were some limitations in our analysis that should be considered. First, the results of any meta-analysis are affected by the quality of the individual studies. Although the included studies were all RCTs, the scores of the studies were not high. Second, the sample size was relatively small in the eligible trials and the scores were not high, which led to the relatively low statistical power of treatment effects, which were evaluated. Therefore, more well-designed and large-scale trials should be conducted in the future. Third, we did not conduct a pooled analysis on survival outcomes because the 3 trials adopted various survival outcome indicators. Survival benefits could not be evaluated accurately. Fourth, because all of the studies included in this meta-analysis were from Asia, the results need confirmation in Western countries.

## CONCLUSION

Our meta-analysis indicated that PTX plus S-1 therapy had near-equivalent safety and a better DCR compared with PTX plus 5-FU therapy. With respect to the quality of life, PTX plus S-1 therapy is a favorable strategy especially for patients who cannot tolerate continuous intravenous infusion; however, more high-quality, large sample-size RCTs and Western studies are needed to confirm these findings.
